# Enhanced cerebrovascular expression of matrix metalloproteinase-9 and tissue inhibitor of metalloproteinase-1 via the MEK/ERK pathway during cerebral ischemia in the rat

**DOI:** 10.1186/1471-2202-10-56

**Published:** 2009-06-04

**Authors:** Aida Maddahi, Qingwen Chen, Lars Edvinsson

**Affiliations:** 1 Department of Clinical Sciences, Division of Experimental Vascular Research, BMC A13, Lund University, 221 84 Lund, Sweden

## Abstract

**Background:**

Cerebral ischemia is usually characterized by a reduction in local blood flow and metabolism and by disruption of the blood-brain barrier in the infarct region. The formation of oedema and opening of the blood-brain barrier in stroke is associated with enhanced expression of metalloproteinase-9 (MMP-9) and tissue inhibitor of metalloproteinase-1 (TIMP-1).

**Results:**

Here, we found an infarct volume of 24.8 ± 2% and a reduced neurological function after two hours of middle cerebral artery occlusion (MCAO), followed by 48 hours of recirculation in rat. Immunocytochemistry and confocal microscopy revealed enhanced expression of MMP-9, TIMP-1, and phosphorylated ERK1/2 in the smooth muscle cells of the ischemic MCA and associated intracerebral microvessels. The specific MEK1/2 inhibitor U0126, given intraperitoneal zero or 6 hours after the ischemic event, reduced the infarct volume significantly (11.8 ± 2% and 14.6 ± 3%, respectively; *P *< 0.05), improved neurological function, normalized expression of phosphorylated ERK1/2, and reduced expression of MMP-9 and TIMP-1 in the vessel walls. Administration of U0126 12 hours after MCAO did not alter the expression of MMP-9. Immunocytochemistry showed no overlap in expression between MMP-9/TIMP-1 and the astrocyte/glial cell marker GFAP in the vessel walls.

**Conclusion:**

These data are the first to show that the elevated vascular expression of MMP-9 and TIMP-1, associated with breakdown of the blood-brain barrier following focal ischemia, are transcriptionally regulated via the MEK/ERK pathway.

## Background

Focal cerebral ischemia results from a reduction in cerebral blood flow to a discrete region of the brain, initiating a complex process that includes release of excitatory neurotransmitters and activation of apoptotic pathways. Even though regional cerebral blood flow is restored to near normal values after two hours of middle cerebral artery occlusion (MCAO) followed by reperfusion [1], a cerebral infarct of about 25% of total brain volume occurs consistently [2]. Some manifestations of the ischemic damage are break-down of the blood-brain barrier (BBB), activation of inflammatory cascades, and disruption of basement membranes and extracellular matrix via cytokine-induced alterations in the expression of metalloproteinases (MMPs) and tissue inhibitor of metalloproteinase-1 (TIMP-1) [3]. MMPs are a family of zinc-binding proteolytic enzymes that can degrade structural proteins of the extracellular matrix (ECM) and cleave other non-ECM molecules ranging from growth factor precursors, cytokines, and binding proteins, to cell surface receptors [4]. In the central nervous system, MMP-9 is involved in disruption of the BBB by degrading tight junction proteins [5]. The proteolytic activity of MMPs is tightly controlled by tissue inhibitors of MMPs (TIMPs)[6]. By degrading the neurovascular matrix, MMPs promote BBB injury, causing brain oedema and haemorrhage. Inhibition of MMP-9 prevents tight junction protein degradation [5], while excessive expression of MMPs contributes to the pathological processes. For example, MMP-2 and MMP-9 are upregulated during cerebral ischemia, however their temporal regulation differs. MMP-9 plays a pivotal role in the degradation of the BBB after focal cerebral ischemia [7] and is also expressed in human brain tissue after ischemic and hemorrhagic stroke [8]. There is an early increase in MMP-9 expression in the microvascular walls after cerebral ischemia and selective inhibition of MMP-9 reduces the brain injury after stroke [9]. MMP-9 peaks at 48 hours while MMP-2 peaks at 5 days post stroke. It has been suggested that the balance between MMPs and TIMP-1 plays a significant role in experimental reperfusion injury [3] and in human stroke [8,10].

In previous studies, we observed rapid transcriptional upregulation of contractile endothelin ET_B _and angiotensin AT_1 _receptors within the cerebrovascular smooth muscle cells in the ischemic region in MCAO-induced focal cerebral ischemia and experimental subarachnoid haemorrhage [11-13]. It is possible that this upregulation promotes the formation of the penumbral damage via enhanced contraction of the vasculature leading to and within the ischemic region, especially considering that the receptor ligands (angiotensin II and endothelin-1) are formed in the cerebrovascular endothelium [14].

Therefore, we examined the early changes in the expression of MMPs and TIMPs, MMP-9 and TIMP-1 in particular. This study demonstrates, for the first time, the enhanced expression of MMP-9 and TIMP-1 after MCAO followed by reperfusion in cerebrovascular smooth muscle cells. Detailed immunocytochemical analysis revealed that this enhanced expression was not associated with other elements of the vessel walls or with glial end-feet or neurons. We asked whether this enhanced expression was associated with activation of mitogen-activated protein kinases (MAPK), a family that includes extracellular signal-regulated kinases (ERK1/2), p38 MAPK, and c-Jun N-terminal kinases (JNK), which transmit extracellular signals into the nucleus to modulate protein expression. Previously, we observed that ERK1/2 was activated early, resulting in cerebrovascular receptor upregulation, while p38 and JNK were activated only after 1–2 days [15,16]. This observation was validated by the results of systemic administration of the specific MEK1/2 inhibitor U0126 [17], which blunted the enhanced activity of the MEK/ERK pathway in the cerebrovascular smooth muscle cells. In addition, we found that MEK1/2 inhibition reduced the infarct size, improved neurological function, and normalized the enhanced expression of MMP-9 and TIMP-1 that follows ischemic injury.

## Results

**Figure 1:**
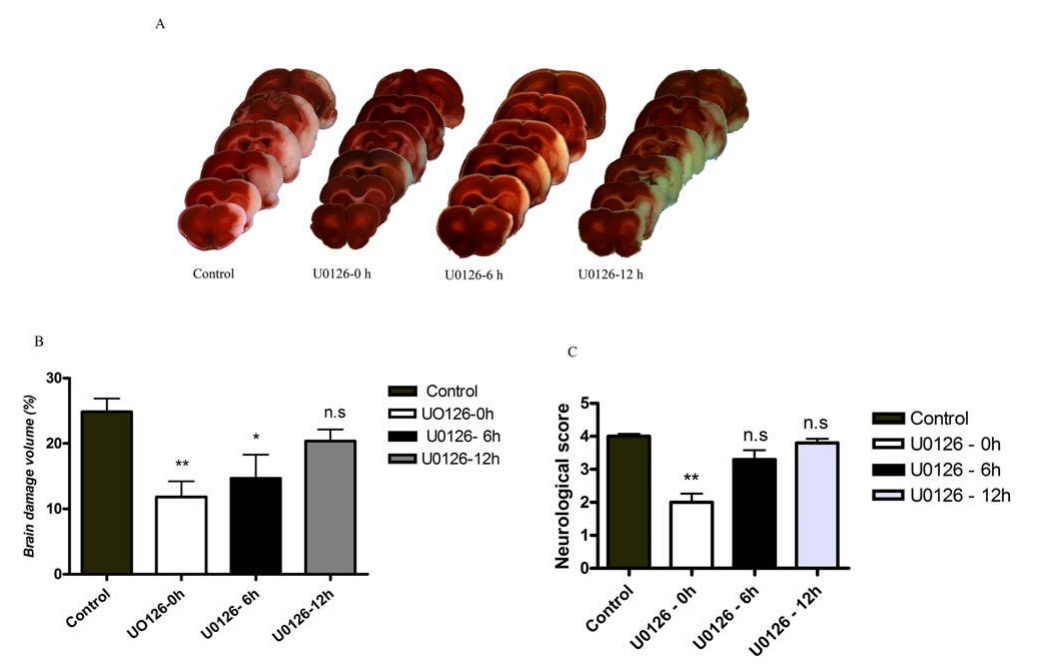
**(A) Following two hours of middle cerebral artery occlusion (MCAO), the area of ischemia in animals treated with U0126 at zero or 6 hours after the start of reperfusion (b and c, respectively) are smaller than the areas area of ischemia in vehicle-treated animals (a; coronal sections stained with 2, 3, 5-triphenyltetrazolium chloride)**. Treatment with U0126 starting at 12 hours after MCAO (**d**) did not decrease the area of ischemia. (**B**) The infarct size (% of total brain volume) was significantly decreased in animals treated with U0126 starting at 0 hours (11.8 ± 2%**) and 6 hours (14.6 ± 3%*) after MCAO as compared to the control group (24.8 ± 2%) and animals treated with U0126 12 hours after MCAO (20.3 ± 1%). (**C**) Neurological assessment scores for U0126-treated rats (0, 6, and 12 hours after MCAO) and vehicle-treated rats (control). Data are expressed as mean ± SEM; n = 6–7. *P < 0.05, ** *P *< 0.01.

In this study, we used the rat model of inducible cerebral ischemia: rats were subjected to reversible MCAO for 2 hours followed by reperfusion for 48 hours [2]. The MCAO produced an occlusion visible by laser Doppler flowmetry as an abrupt 80–90% reduction in local cortical blood flow that normalized after removal of the occluding thread (that was not different from before occlusion in the operated rats). There were no significant differences in physiological parameters between the different treatment groups for blood pressure, blood gases, temperature, plasma glucose, and body weight (data not shown). Following rapid sacrifice, we collected tissue for immunocytochemistry, western blot, and calculation of infarct volume (24.8 ± 2% of total cerebrum in the MCAO group; Fig. 1A and 1B). Neurological evaluations were performed just before animal sacrifice (MCAO group, 4.0 ± 0.2 versus sham-operated animals with no visible defects resulting in a score of 0; *P *< 0.05; Fig. 1C).

### Analysis of infarct volume, neurological examination, and vessel wall protein expression

Previously, immunocytochemical and western blot analyses showed that MCAO with reperfusion caused activation of the MEK/ERK pathway in cerebral vessels associated with the ischemic region [18]; data from our study confirm this observation. Firstly, intravenous administration of the MEK1/2 inhibitor U0126 at 0 or 6 hours after the two-hour MCAO and initiation of reperfusion significantly reduced the infarct volume (11.8. ± 2% and 14.6 ± 3%, respectively; *P *< 0.05; Fig. 1A and 1B) and improved neurological assessment scores (2 ± 0.7 and 3.3 ± 0.7, respectively; *P *< 0.05; Fig. 1C). When U0126 treatment was initiated 12 hours after the start of reperfusion, there was no significant reduction in infarct volume or neurological score as compared to control animals (21 ± 2% and 3.8 ± 0.8%, respectively; *P *< 0.05; Fig. 1). Secondly, after MCAO, pERK1/2 activity in the vascular smooth muscle cells was upregulated in large cerebral arteries (218 ± 11%; *P *< 0.05) and in microvessels (130 ± 10%; *P *< 0.05) but not in adjacent brain tissue (103 ± 4%; *P *> 0.05), as previously shown [18]. U0126 treatment initiated at zero or 6 hours after initiation of reperfusion normalized vascular pERK1/2 expression (108 ± 5% and 106 ± 7%, respectively; *P *< 0.05).

### Expression of MMP-1 and TIMP-1

**Figure 2:**
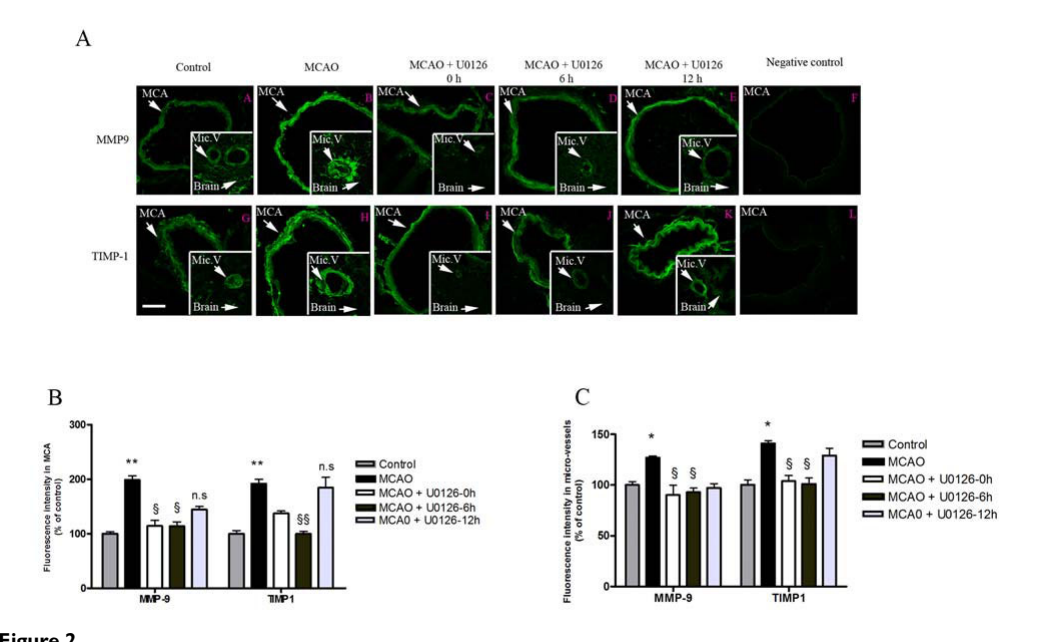
**(A) Confocal microscopy images of the ischemic middle cerebral artery (MCA), cerebral micro-vessels (Mic.V), and surrounding brain tissue (Brain) immunofluorescently labeled for MMP-9 (a-f) or TIMP-1 (g-l)**. Images represent the vehicle control group (**a**, **g**), MCAO plus vehicle group (**b**, **h**), MCAO plus U0126 starting at 0 hours (**c**, **i**), 6 hours (**d**, **j**), or 12 hours (**e**, **k**) groups, and the negative control group (**f**, **l**). There was a significant increase in the MMP-9 protein level in the smooth muscle cell layer of ischemic vessels (MCA and Mic.V) as compared with vessels from the vehicle control group. TIMP-1 expression was upregulated in smooth muscle cells and in the proximity of the adventitia layer of ischemic vessels as compared to control vessels. Treatment with U0126, starting at zero and 6 hours, but not at 12 hours, after occlusion prevented the increase in MMP-9 and TIMP-1 protein expression. There was a slight increase in MMP-9 protein expression in ischemic brain tissue and in astrocytes around the vessels as compared to control and U0126-treated brain tissue. For TIMP-1, was no difference in protein expression in control brain tissue, in ischemic brain tissue, or tissue from animals treated with U0126. Scale bar, 50 μm. **(B, C) **Bar graphs showing the fluorescence intensity for MMP-9 and TIMP-1 in the MCA and micro-vessels. There was a significant increase in MMP-9 and TIMP-1 protein expression in MCAO animals as compared to control animals; this increase was prevented with U0126 treatment starting at zero and 6 hours, but not 12 hours, post MCA. Data are presented as the mean percentage relative to control ± SEM.; *n *= 5. *P < 0.05, **P < 0.01.

**Figure 3:**
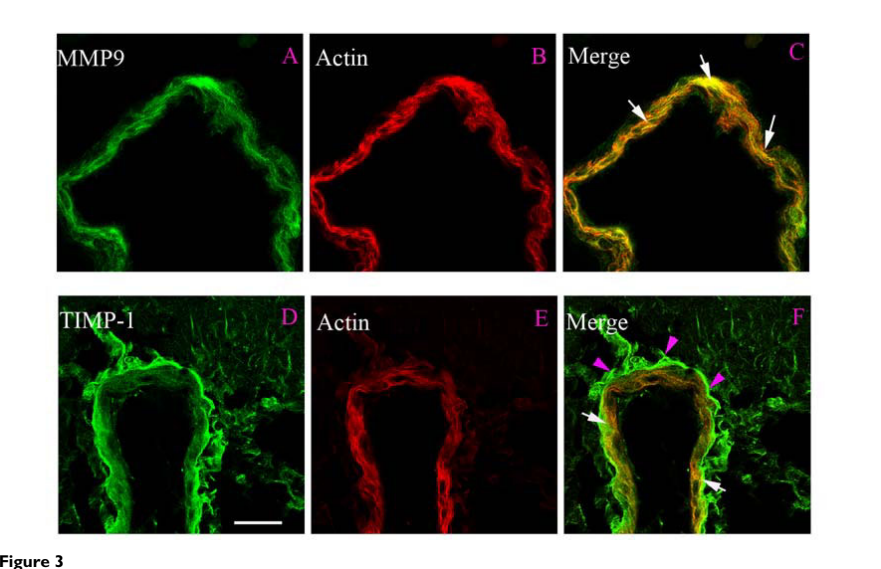
**Double immunofluorescence staining for MMP-9 or TIMP-1 and actin in smooth muscle cells of the middle cerebral artery after MCAO**. (**A**-**C**) Photographs demonstrating the localization of MMP-9, actin immunostaining, and their co-localization in smooth muscle cells (yellow fluorescence in the merged picture). (**D**-**F**) TIMP-1 immunostaining, actin immunostaining, and their co-localization in the smooth muscle cells (white arrows). Scale bar, 50 μm.

**Figure 4:**
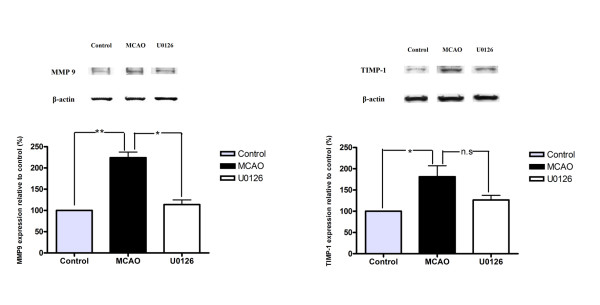
**Immunoblots showing MMP-9 and TIMP-1 protein expression levels in the middle cerebral artery 48 hours after MCAO using β-actin as a loading control**. Treatment with U0126 at 0 hours post occlusion decreased the MCAO-induced enhanced expression of MMP-9 and TIMP-1 receptor proteins. Data from four experiments (each performed on vessels from 3 rats) are expressed as mean ± SEM, n = 4. *P < 0.05, **P < 0.01.

Subsequently, we examined the MCA, cerebral microvessels, and the surrounding brain tissue in the ischemic region and on the contralateral side for changes in expression of MMP-9 and TIMP-1 protein at 48 hours post MCAO. We found markedly enhanced expression of MMP-9 in the vascular smooth muscle cells from the ischemic region; the expression was localized to the cytoplasm, leaving the nuclear regions clear of MMP-9 immunoreactivity (Fig. 2A). TIMP-1 expression was observed in the media layer, but was located closer to the adventitia layer of the cerebral vessel walls and hence only to some degree in the smooth muscle cells (Fig. 2A). Quantitative evaluation of the expression levels revealed significant upregulation of MMP-9 and TIMP-1 after MCAO in the MCA and in the microvessels, while only faint staining was seen in vehicle-treated animals (Table 1, Table 2, Fig. 2B and 2C). Results from double immunostaining for MMP-9 or TIMP-1, and actin revealed that the expression of these proteins was localized to the smooth muscle cells in the MCA and cerebral microvasculature (Fig. 3); however, their distributions varied slightly (see below). CD31 was used as a marker of endothelial cells; neither MMP-9 nor TIMP-1 revealed any major co-localization with CD31 (data not shown), hence the upregulation occurred in the media layer. The results from western blot experiments of MCAs (n = 4 in each group, with 3 animals pooled for each measurement) showed that the protein levels of MMP-9 and TIMP-1 were significantly increased after MCAO as compared to vehicle-treated animals (224 ± 13% and 181 ± 25%, respectively; *P *< 0.05; Fig. 4). Administration of the MEK1/2 inhibitor U0126 immediately after the initiation of reperfusion (0 hour) decreased the levels of MMP-9 and TIMP-1 proteins by 113 ± 11% and 126 ± 10%, respectively (Fig. 4).

**Table 1:** Levels of MMP-9 and TIMP-1 protein in the middle cerebral artery after MCAO and after treatment with the MEK1/2 inhibitor U0126 at 0, 6, and 12 hours post occlusion

Middle cerebral artery					
	Control	MCAO	MCAO + U0126 - 0 h	MCAO + U0126 - 6 h	MCAO + U0126 - 12 h
					
MMP-9 (%) ± SEM	100 ± 3	198 ± 7**	114 ± 10^§^	113 ± 8^§^	144 ± 5
					
TIMP-1 (%) ± SEM	100 ± 5	191 ± 7**	137 ± 5	100 ± 4^§§^	184 ± 19

**Table 2:** Levels of MMP-9 and TIMP-1 protein in the microvessels after MCAO and after treatment with U0126 at 0, 6, and 12 hours post occlusion

Cerebral microvessels					
	Control	MCAO	MCAO + U0126 - 0 h	MCAO + U0126 - 6 h	MCAO + U0126 - 12 h
					
MMP-9 (%) ± SEM	100 ± 3.5	127 ± 1.5*	90 ± 9.8^§^	92.5 ± 4.05^§^	97 ± 4.4
					
TIMP-1 (%) ± SEM	100 ± 5	141 ± 2.6*	104 ± 5.3^§^	101 ± 6^§^	129 ± 7.3

### Association with astrocyte end-feet

**Figure 5:**
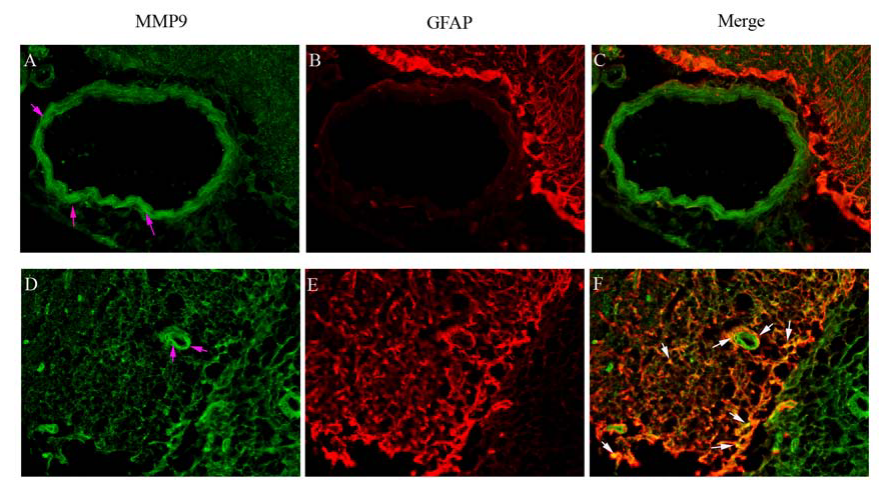
**Double immunofluorescence staining for MMP-9 and GFAP demonstrate their localization in the middle cerebral artery (MCA), microvessels, and in surrounding brain tissue**. MMP-9 expression in the smooth muscle cells of the MCA, micro-vessels, and brain tissue after MCAO is shown in (**A**) and (**D**) (pink arrows). GFAP expression was observed in astrocytes around the MCA, microvessels, and in the brain tissue (**B **and **E**). The merged images show the co-localization of MMP-9 and GFAP (**C **and **F**). There was no co-localization between MMP-9 and GFAP in the MCA (**C**), but there was some co-localization between MMP-9 and GFAP in the brain tissue and modest co-localization in the astrocytic end-feet surrounding the microvessels (white arrows).

**Figure 6:**
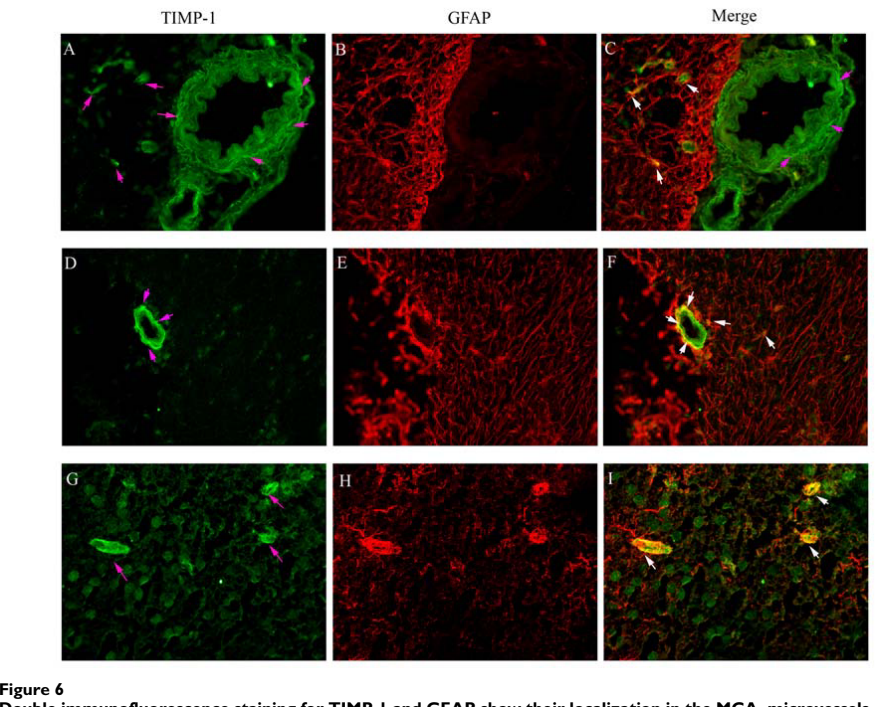
**Double immunofluorescence staining for TIMP-1 and GFAP show their localization in the MCA, microvessels, and brain tissue**. TIMP-1 expression was seen in the cell walls of the MCA and microvessels and in nerve cells and astrocytes in the brain tissue (pink arrows; **A**, **D**, and **G**). GFAP expression was observed in the astrocyte cells around the MCA and microvessels and in the brain tissue (**B**, **E**, and **H**). Illustration of the co-localization of TIMP-1 and GFAP expression (**C**, **F**, and **I**). TIMP-1 immunoreactivity co-localized with GFAP immunoreactivity in the nerve cells and some areas of astrocytes in the brain tissue (**C**) and at the outer layer of microvessels; these areas were surrounded by astrocytic end-feet (white arrows; **F **and **I**).

GFAP is a selective marker of astrocytes, which are known to be intimately associated with cerebral microvasculature [19]. We detected no GFAP-immunopositive end-feet in the walls of the MCA (or other major cerebral arteries belonging to the circle of Willis) but confirmed that there is a rich network of GFAP-positive astrocytes in the cerebral cortex tissue (Fig. 5 and 6). Here, the astrocytic end-feet surrounded the microvasculature, as previously described[20]. MMP-9 immunoreactions in the MCA and the microvessels were clearly dissociated from GFAP-positive staining at all time points studied. However, in the microvessels (Fig. 5), the astrocytic end-feet closely encircled the vessel walls and came adjacent to the smooth muscle cells but only in the outermost part of the media layer, showing a slight merging under confocal microscopy. The situation for TIMP-1 was different; TIMP-1 immunoreactivity was mainly present in the outer part of the media layer and in the adventitia of the cerebral vessels, still closely associated with the smooth muscle cells, as demonstrated in co-localization studies with actin. In this part of the vessel walls MMP-9 and TIMP-1 co-located (data not shown). In microvessels, the association with astrocytic end-feet was more intimate because both GFAP and TIMP-1 immunoreactivity occurred in the outermost part of the media and in the adventitia, sometimes appearing merged in the walls of the microvessels (Fig. 6).

### Inhibition of MEK1/2 activity in vivo

Next, we assessed whether the MEK/ERK pathway was activated in the walls of the MCA, the microvessels, and surrounding brain tissue following MCAO. Results from immunostaining with pERK1/2-specific antibodies showed that pERK1/2 expression in the smooth muscle cells in the vasculature was significantly increased in the ischemic region at 48 hours post MCAO (174 ± 6%; *P *< 0.05). Systemic administration of the MEK1/2-specific inhibitor U0126 [21] either immediately after release of the occlusion (0 hour) or 6 hours post MCAO recirculation effectively abolished the increase in pERK1/2 activity in the ischemic MCA and the cerebral microvessels (see above). However, there was no visible alteration in pERK1/2 activity in brain tissue of the ischemic (117 ± 13%; *P *> 0.05) or contralateral regions (data not shown).

Treatment with U0126 (beginning at 0 and 6 hours post MCAO) significantly decreased the upregulation of MMP-9 and TIMP-1 in both the MCA and the cerebral microvessels within the infarct area (Table 1 and 2) but no difference in brain tissue per se (Table 3). However, administration of U0126 beginning 12 hours after reperfusion did not significantly reduce the ischemia-induced expression of MMP-9 or TIMP-1 in the cerebral vessel smooth muscle cells (Table 1 and 2). These results were confirmed at the protein level by western blot.

**Table 3:** Levels of MMP-9 and TIMP-1 protein in brain tissue after MCAO and after treatment with U0126 at 0, 6, and 12 hours post occlusion

Brain tissue					
	Control	MCAO	MCAO + U0126 - 0 h	MCAO + U0126 - 6 h	MCAO + U0126 - 12 h
					
MMP-9 (%) ± SEM	100 ± 5.8	125 ± 8.0	101 ± 6.5	102 ± 4.7	109 ± 7.2
					
TIMP-1 (%) ± SEM	100 ± 3.8	113 ± 2.7	111 ± 5	105 ± 2.7	112 ± 5.2

Values are percentages of the control value and are reported as mean ± standard error of the mean (SEM); n = 5–6, *P < 0.05, **P < 0.01, §*P *< 0.05, §§ *P *< 0.01. *Indicates a significant difference between the control group and the MCAO group; § indicates a significant difference between the MCAO group and the U0126-treated group.

## Discussion

This is the first study to clearly demonstrate that two-hour MCAO followed by 48 hours of reperfusion results in significant upregulation of MMP-9 and TIMP-1 in the smooth muscle cells of the MCA and in microvessels within the ischemic region. Furthermore, our data show that this upregulation is associated with upregulation of pERK1/2 and normalized by inhibition of the MEK/ERK pathway.

To determine the cellular source of MMP-9 and TIMP-1, we performed confocal microscopy and co-localization studies using smooth muscle actin-specific antibodies. MMP-9 immunoreactivity was localized to the cytoplasm of the cerebral vessel smooth muscle cells, both in the MCA and in intracerebral microvessels. Although minor amounts of actin has been observed in endothelial cells we could easily dissociate microscopically the endothelium from the smooth muscle cells as they are separated by an inner elastic lamina [22]. In addition, some vessels were studied after mechanical removal of the endothelium. After this procedure the localization of the immunoreactions to the smooth muscle cells was still confirmed (data not shown). This increase in immunoreactivity agrees with a previously reported increase in MMP-9 mRNA and protein expression in the ischemic tissue at 24 hours after MCAO (without any change in MMP-2 mRNA), and this correlated with opening of the BBB [5]. These investigators observed that MMP-2 co-localized with GFAP-expressing astrocytes and with neurons in the lateral and piriform cortices, but not in the vessel walls [5]. It was also shown that increased MMP-9 activity was associated with a reduction in junction proteins in cerebrovascular endothelial cells and in BBB disruption after focal ischemia [23]. Detailed analysis revealed that these events were caused by MMP-9-mediated degradation of the junction proteins claudin-5 and occludin. In support of these data, the administration of an MMP-9 blocker prevented this degradation and abolished the BBB damage [5].

There exist some data on the time-dependency of the elevation in expression of MMP-9 in the cerebral vessel walls. Thus, the direct comparison of MMP-9 expression in the present ischemic model with that seen in experimental subarachnoid haemorrhage (SAH) and after organ culture of isolated MCA segments revealed enhanced levels of MMP-9 mRNA at 6 and 24 hrs [24]. The time-course was studied in more detail after experimental SAH; the main expression of MMP-9 was seen at 48 hours [25].

The specific MEK1 inhibitor U0126 does not affect phosphorylation of p38 or JNK in cultured neurons [17] or in cerebrovascular smooth muscle cells *in vivo *using the present model of ischemia [24]. Detailed western blot experiments have confirmed the specificity of U0126 on the MEK/ERK pathway [26]. Therefore, we can rule out that U0126 acts via non-specific inhibition of the pro-apoptotic and pro-inflammatory mechanisms since unknown non-MEK effects cannot be ruled out. U0126 has been found to increase MEK1/2 phosphorylation in cortical neurons, thus U0126 does not affect components upstream of MEK1/2 [27]. Hence, it is reasonable to assume that the neuroprotective effect of U0126 results from the inhibition of cerebrovascular MEK1/2 activity, which agrees with the observed reductions in the activity of the downstream MAPK, pERK1/2. In this study, we showed that MCAO resulted in enhanced expression of pERK1/2 in smooth muscle cells of the ischemic MCA and associated microvessels (shown by co-localization with actin) but not in the surrounding brain tissue; U0126 blunted this activation, reduced the infarct volume, and improved the neurological assessment scores of treated rats. Intriguingly, inhibiting this sequence of events correlated with the inhibition of MMP-9 and TIMP-1 expression in the same location. Quantitative real time-PCR demonstrated enhanced mRNA expression of MMP-9 24 hours after MCAO in cerebral blood vessels in focal ischemia, and at 24 and 48 hours after experimental SAH [24]. Our data indicate, for the first time, that the expression of MMP-9 and TIMP-1 in cerebral blood vessel smooth muscle cells is enhanced after cerebral ischemia and that this enhancement is a transcriptional event. While constitutively expressed MMP-2 is involved in an early brief loosening of tight junctions and the initial reversible opening of the BBB, MMP-9 expression increases with time, is more durable, and is possibly related to increased neuroinflammation [5]. Importantly, the opening of the BBB is associated with brain damage and our observations reveal a mechanism by which to modify the expression of MMP-9, thereby reducing the risk of brain damage: inhibiting MEK1/2.

Although MEK/ERK-pathway mechanisms play a crucial roles in mediating brain injury after ischemia and reperfusion, and inhibiting this pathway can reduce the infarct size [17,28], we provide direct evidence supporting an explanation for some of the events related to the focal pathology of cerebral ischemia [29]. U0126 administration diminished pERK1/2 immunoreactivity in the ischemic brain of the mouse [17] and in the MCA of the rat [28]. In the mouse model, three hours of MCAO was followed by 24 hours of reperfusion. Interestingly, the infarct volume was affected only if U0126 was given in conjunction with the MCAO [17]. Furthermore, in a permanent MCAO model, pre-treatment with U0126 was necessary to inhibit pMEK1/2/pERK1/2 expression *in vivo *in the mouse brain in both the ischemic core and perifocal regions [30]. Also, the specificity of the antagonism revealed that U0126 does not inhibit the cellular synthesis of ERK1/2 but does block the ERK1/2 phosphorylation and activation of, for example, the transcription factor ELK-1 [30,31]. In agreement with our observations, MEK1/2 inhibition does not alter cortical blood flow within the first few hours of administration [17,32] or modify the contractility of isolated cerebral arteries (unpublished data). Hence, U0126 does not act on the cerebral circulation through a direct vasodilator mechanism. Instead, we suggest that U0126 blunts receptor upregulation [28].

Here, we have demonstrated yet another positive effect of U0126: blocking the enhanced expression of MMP-9, which participates in the destruction of the BBB and subsequent brain oedema. More importantly, systemic administration of U0126 markedly reduced the infarct size and improved neurological function, even when the first dose was given as late as 6 hours after the initiation of reperfusion. Clearly, these data differ to some degree from those found in previous reports. While U0126 is a well-known inhibitor of MEK1/2, it has a questionable permeability of the BBB. In order to overcome this hurdle, we used a much higher dosage of U0126 (30 mg/kg) than that used by others, which still did not alter any physiological parameters and was well tolerated by the rats. This dosage is much higher than that necessary to inhibit the MEK/ERK pathway in cell culture but is necessary to allow a sufficient dosage to reach the abluminal side of the BBB; this was verified by protein expression using both immunohistochemistry and western blot. We verified the success of the antagonism by demonstrating that MCA and cerebral microvascular pERK1/2 activities were reduced to control levels and this reduction was associated with a significant reduction in infarct size and reduced expression of MMP-9 and TIMP-1. If U0126 treatment was initiated 12 hours after the start of reperfusion, there was no significant effect on the above parameters. Therefore, at this dosage, the MEK1 inhibitor U0126 may have a therapeutic window.

## Conclusion

We hypothesise that MEK/ERK inhibition might represent a way to prevent stroke-induced pathology because it targets several transcriptional mechanisms activated by cerebral ischemia, such as receptor upregulation, which causes enhanced contractility, and MMP-9 and TIMP1 activation, which affect the function of the BBB. Inhibition of the MEK/ERK pathway applied as late as 6 hours after the start of reperfusion significantly reduced the infarct volume and the expression of BBB-associated proteins MMP-9 and TIMP1 in the cerebral vessel walls. Thus, the positive effects of MEK/ERK inhibition might involve several mechanisms in the MCA and in brain microvasculature associated with the cerebral ischemia. However, the dosage required to reach targets on the abluminal side of the BBB is a factor that warrants further study.

## Methods

### Middle cerebral artery occlusion

A total of 56 Male Wistar-Hanover rats (Møllegaard Breeding Centre, Copenhagen, Denmark) weighing approximately 300–350 g were obtained from Harlan, Horst, Netherlands, and were used for the procedures. The animals were housed under controlled temperature and humidity with free access to water and food. The experimental procedures were approved by the University Animal Ethics Committee (M43-07).

Anaesthesia was induced using 4.5% halothane in N_2_O:O_2 _(70%:30%) and was maintained by inhalation of 1.5% halothane by mask. To confirm proper occlusion of the right MCA, a laser-Doppler probe (Perimed, Järfälla, Sweden) was fixed on the skull (1 mm posterior to the bregma and 6 mm from the midline on the right side) to measure local cortical blood flow in an area supplied by the MCA. A polyethylene catheter was inserted into a tail artery to measure the mean arterial blood pressure, pH, pO_2_, pCO_2_, and plasma glucose. A rectal temperature probe connected to a homeothermal blanket was used to maintain body temperature at 37°C during the procedure.

An intraluminal filament technique was used to induce transient MCAO [2]. Briefly, an incision was made in the midline of the neck and the right common, external, and internal carotid arteries were exposed. The common and external carotid arteries were permanently ligated with sutures. A filament was inserted into the internal carotid artery via an incision in the common carotid artery and advanced until the rounded tip reached the entrance to the right MCA. The resulting occlusion was visualized by laser-Doppler as an abrupt 80–90% reduction in local cortical blood flow After 2 h of occlusion, the rat was re-anesthetized to allow withdrawal of the filament; reperfusion was verified by laser-Doppler recording. 20–30% of the animals were terminated in conjunction with surgery and MCAO due to imperfect drop in laser-Doppler flow. (They were not included because they did not show the expected reduction in blood flow).

### Treatments

To inhibit MEK1/2, animals were injected intraperitoneal with 30 mg/kg/day of U0126 dissolved in dimethylsulfoxide (DMSO), beginning at reperfusion (0 h), at 6 h, 12 h, or 24 h post occlusion [33]. Rats in the control groups were injected with equal volumes of DMSO. The dose of U0126 was chosen on the basis of previous experiments [18].

### Harvesting cerebral vessels and brain tissue

At 48 h post MCA occlusion, MCAO rats, and MCAO rats treated with U0126, and their respective DMSO controls were anesthetized and decapitated. The brains were removed and immersed in ice-cold bicarbonate buffer solution of the following composition (mM): NaCl 119, NaHCO_3_, 15, KCl 4.6, MgCl_2 _1.2, NaH_2_PO_4 _1.2, CaCl_2 _1.5, and glucose 5.5. The right and left MCAs and surrounding brain tissue were dissected out using a dissection microscope, snap frozen, and stored at -80°C for immunohistochemical analysis. A large number of proximal MCA segments were also harvested and pooled for protein extraction and western blot analysis.

### Neurological examination

The animals were subjected to a neurological examination prior to recirculation and immediately before they were sacrificed (48 hours after MCAO), according to an established scoring system [34,35]: 0, no visible deficit; 1, contralateral forelimb flexion, when held by tail; 2, decreased grip of contralateral forelimb; 3, spontaneous movement in all directions, but contralateral circling if pulled by tail; 4, spontaneous contralateral circling; 5, death.

### Brain damage evaluation

The brains were sliced coronal in 2-mm thick slices and stained with 0.5 mg/ml 1% 2, 3, 5-triphenyltetrazolium chloride (Sigma, St Louis, MO) dissolved in buffer solution at 37°C for 20 minutes. The extent of the ischemic brain damage was calculated as a percentage of the total brain volume in the slices using the software program Brain Damage Calculator 1.1 (MB Teknikkonsult, Lund, Sweden). The pictures were evaluated by two independent researchers unknown to the treatment group.

### Immunofluorescence

For immunofluorescence analysis, the MCA and the surrounding brain tissue were dissected out, placed into Tissue TEK (Gibo, Invitrogen A/S, Taastrup, Denmark), and frozen on dry ice; thereafter, they were sectioned into 10-μm thick slices. Cryostat sections of the arteries and brain tissue were fixed for 10 minutes in ice-cold acetone (-20°C) and then rehydrated in phosphate buffer solution (PBS) containing 0.3% Triton X-100 for 15 minutes. The tissues were then permeabilized and blocked for 1 hour in blocking solution containing PBS, 0.3% TritonX-100, 1% bovine serum albumin (BSA), and 5% normal donkey serum, and then incubated over night at 4°C with either rabbit anti-phosphoERK1/2 MAPK (ab4376; Cellsignaling, Danvers, MA) diluted 1:50, rabbit anti-rat MMP-9 (ab7299; Abcam, Cambridge, MA) diluted 1:400, or rabbit anti-human TIMP-1 (AB770; Chemicon, Copenhagen, Denmark) diluted 1:200. All primary antibodies were diluted in PBS containing 0.3% Triton X-100, 1% BSA, and 2% normal donkey serum. Sections were subsequently incubated for 1 hour at room-temperature with secondary Cy™^2^-conjugated donkey anti-rabbit (711-165-152; Jackson ImmunoResearch, Europe Ltd., Suffolk, UK) diluted 1:200 in PBS containing 0.3% Triton X-100 and 1% BSA. The sections were subsequently washed with PBS and mounted with Permafluore mounting medium (Beckman Coulter, PNJM0752). Immunoreactivity was visualized and photographed using a Nikon confocal microscope (EZ-c1, German) at the appropriate wavelength. The same procedure was used for the negative controls except that primary or secondary antibodies were omitted. There was also a know sample as positive control to compare with the present samples to avoided any probability failure in results. Data using blocking peptide (sequence used for the immunization) were provided by the supplier.

### Double immunofluorescence

Double immunofluorescence labelling was performed for TIMP-1, MMP-9, and phosphorylated ERK1/2 versus smooth muscle actin or glial fibrillary acidic protein (GFAP), an astrocyte/glial cell marker. In addition to the antibodies described above, we used mouse anti-rat smooth muscle actin antibodies (SC-53015; Santa Cruz Biotechnology, Inc, Santa Cruz, CA) diluted 1:200 and mouse anti-GFAP (G3893; Sigma) diluted 1:600 in PBS containing 0.3% Triton X-100, 1% BSA, and 2% normal donkey serum. The secondary antibodies were Cy™^2^-conjugated donkey anti-rabbit (Jackson ImmunoResearch) diluted 1:200 and Texas Red-labeled donkey anti-mouse (Jackson ImmunoResearch Europe) diluted 1:300 in PBS containing 3% Triton X-100 and 1% BSA. The antibodies were detected at the appropriate wavelengths using a confocal microscope (EZ-cl, Germany).

### Image analysis

Fluorescence intensity was measured using ImageJ software . Measurements were made in 4 different preset areas (located on the clock at 0, 3, 6 and 9 h) from 4 vessel sections from each vessel sample and the investigator was blinded to the treatment group of each sample. The fluorescence intensity of each treatment group was given as the percentage change relative to control; the control value was normalized to 100%. The mean value for each was used for comparisons [33,36].

### Western blotting

Proximal MCA segments (n = 12 rats in each group; vessels from 3 rats were pooled for each measurement and experiments were done in total 4 times) were harvested and frozen in liquid nitrogen and homogenized in cell extract denaturing buffer (BioSource, Carlsbad, CA) that contained both phosphatase inhibitor and protease inhibitor cocktails (Sigma). Whole cell lysates were sonicated on ice for 2 min, centrifuged at 15 000 × g at 4°C for 30 min, and the supernatants were collected as protein samples. Protein concentrations were determined using standard protein assay reagents (Bio-Rad, Hercules, CA) and stored at -80°C awaiting immunoblot analysis. The protein homogenates were diluted 1:1 (v/v) with 2× sodium dodecyl sulfate (SDS) sample buffer (Bio-Rad). Protein samples (25–50 μg of total protein) were boiled for 10 min in SDS sample buffer and separated on 4–15% SDS Ready Gel Precast Gels (Bio-Rad, USA) for 120 min at 100 v and transferred to nitrocellulose membranes by electroblotting (Bio-Rad) at 100 v for 60 min. The membrane was then blocked for 1 hour at room temperature with PBS containing 0.1% Tween-20 (Sigma) and 5% non-fat dried milk and incubated with primary antibodies, as appropriate [rabbit anti-rat MMP-9 (ab7299; Abcam) and rabbit anti-human TIMP-1 (AB770; Chemicon)], diluted 1:200-1 000 overnight at 4°C, followed by incubation with horseradish peroxidase (HRP)-conjugated anti-rabbit IgG secondary antibodies (Amersham Biosciences, Piscataway, NJ) diluted 1: 5 000–10 000 for 1 hour at room temperature. The labeled proteins were developed using the LumiSensor Chemiluminescent HRP Substrate kit (GenScript Corp., Piscataway, NJ). To detect multiple signals on a single membrane, the membrane was incubated in Restore Plus western blot stripping buffer for 5–15 min at room temperature (Pierce Biotechnology, Inc., Rockford, IL) between the various labeling procedures. The membranes were visualized using a Fujifilm LAS-1000 Luminescent Image Analyzer (Stamford, CT), and band intensity was quantified using Image Gauge Version 4.0 (Fuji Photo Film Co., Ltd., Japan). Three independent experiments were performed in duplicate.

### Calculations and statistical analyses

Data are expressed as the mean ± standard error of the mean (SEM). Statistical analyses were performed using the nonparametric Kruskal-Wallis test with Dunn's post hoc test for quantitative immunohistochemistry and western blot evaluation. One-way analysis of variance (ANOVA) with Dunnett's test was used for infarct volume studies (using GraphPad Prism v 4). *P*-values less than 0.05 were considered significant; "n" refers to the number of rats.

## Authors' contributions

All authors read and approved the final manuscript. AM carried out the main part of the experiments, participated in the design, statistical analysis and writing of the manuscript. QC performed the western blot experiments and participated in the writing of the manuscript. LE conceived the study, directed the work, and drafted the manuscript.
